# Full-parameter-modulated three-dimensional vectorial generalized vortex array

**DOI:** 10.1038/s41377-025-02065-9

**Published:** 2026-01-01

**Authors:** Xue Zhang, Yang Cui, Yanjie Chen, Xiaowei Li, Junjie Li, Wenqiao Shi, Jian Chen, Zhaogang Dong, Yongtian Wang, Cheng-Wei Qiu, Shuang Zhang, Lingling Huang

**Affiliations:** 1 Beijing Engineering Research Center of Mixed Reality and Advanced Display, School of Optics and Photonics, Beijing Institute of Technology, Beijing, 100081 China; 2 Institute of Photonic Chips, University of Shanghai for Science and Technology, Shanghai, 200093 China; 3 Laser Micro/Nano-Fabrication Laboratory, School of Mechanical Engineering, Beijing Institute of Technology, Beijing, 100081 China; 4 Beijing National Laboratory for Condensed Matter Physics, Institute of Physics, Chinese Academy of Sciences, Beijing, 100191 China; 5 Department of Electrical & Computer Engineering, National University of Singapore, Kent Ridge, Singapore, 117583 Singapore; 6 School of Optical-Electrical and Computer Engineering, University of Shanghai for Science and Technology, Shanghai, 200093 China; 7 Science, Mathematics, and Technology (SMT), Singapore University of Technology and Design (SUTD), 8 Somapah Road, Singapore, 487372 Singapore; 8 Quantum Innovation Centre (Q.InC), Agency for Science Technology and Research (A*STAR), 2 Fusionopolis Way, Innovis #08-03, Singapore, 138634 Republic of Singapore; 9 Department of Physics, University of Hong Kong, Hong Kong, China

**Keywords:** Micro-optics, Fibre optics and optical communications, Nanophotonics and plasmonics

## Abstract

Orbital angular momentum, as an important spatial degree of freedom of light, has prompted various promising applications. The recently proposed generalized vortex beams may further enhance the flexibility by utilizing customer-defined angular phase gradients, enabling intuitive graphic representation of mathematical operations and other interesting functionalities. Here, based on Dammann optimization, we propose and demonstrate a three-dimensional generalized vortex beam array using a single-layer metasurface, with all-parameter modulation including polarization, phase, angular momentum, and stereoscopic space. Furthermore, simultaneous vectorial modulation within each order can be endowed through joint optimization to achieve arbitrary polarization information distribution. This novel approach to generating the 3D generalized vortex beam array offers great flexibility in utilizing multiple degrees of freedom of light, further expanding the information capacity and spatial mode features and facilitating applications such as optical wireless broadcasting, optical communication encryption, structured beam manipulation, etc.

## Abstract (web summary)

We demonstrate a 3D generalized vector vortex array generation with full polarization, phase, OAM, and spatial control, by using joint optimization in different diffraction orders based on a single-layer metasurface.

## Graphical abstract

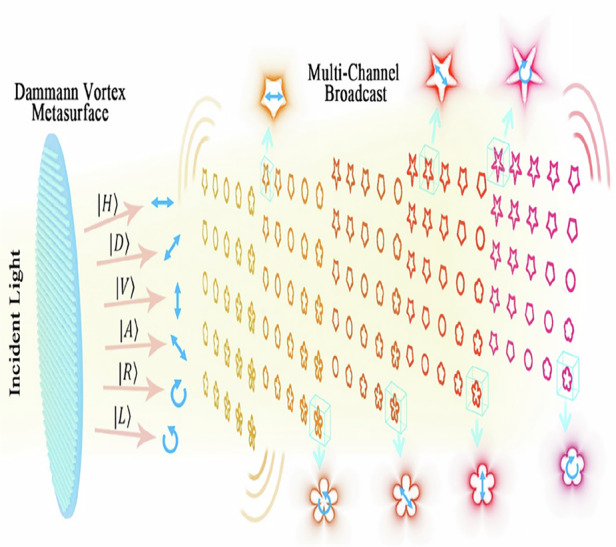

## Introduction

Metasurfaces provide a revolutionary platform for wavefront modulation^1,2^, owing to their flexibility in manipulating phase^3^, amplitude^4^, polarization^5^, frequency^6–8^ and angular spectrum^9^ through tailoring the geometry and arrangement of meta-atoms array^10–13^. When combined with intelligent algorithms, metasurfaces can achieve novel optical effects such as vectorial display^14,15^, orbital angular momentum (OAM) multiplexing/demultiplexing^16^, holography^17^, structured beams^18^ and Integrated systems^19,20^. Compared to traditional optical devices, metasurfaces offer significant advantages in jointly modulating multidimensional light information^21–23^. Their ultracompact integration capability enables the creation of minimized meta-devices, such as light source, spectrometer^24^, polarization camera^25^, and microscope^26^. Additionally, the large space-bandwidth product of metasurfaces promises extensive storage or display capacity of wavefront^27,28^. In particular, the exploration of diffraction orders for information channels surpasses traditional gratings and diffractive optical elements due to the abilities of phase and even polarization manipulation^29^. Various applications have been developed, including multi-order vectorial holographic display^30,31^, parallel all-optical computing^32,33^, switchable multi-mode diffraction^29^, and optical tweezers^34^. However, most of the demonstrations are limited to one-dimensional or two-dimensional arrays^35,36^, and the investigation of spatially variant three-dimensional (3D) arrays remains largely unexplored.

Vortex beams, carrying OAM with phase singularity of exp(*ilφ*), feature donut shape intensity profiles. Vortex arrays are widely used as optical information carriers^37^ due to the orthogonality property of OAM through spatial multiplexing techniques. Dammann vortex metasurfaces^38,39^ have been used to realize the multiplexing and demultiplexing of OAM information^40^, and a 3D vortex array has been demonstrated to carry the spatially variant topological charge distribution^41^. By introducing nonlinear azimuthal phase profile to shape the intensity profile^42^, the generalized vortex beam (GVB) introduces new degrees of freedom to vortex beams. This breaks the rigid impression of a traditional vortex beam with a constant phase gradient while still maintaining phase singularity and angular momentum conservation properties. Furthermore, by generating a GVB array through Taylor expansion with a metasurface, it is possible to perform intuitive mathematical calculations of differential adder and subtractor in different diffraction orders^43^.

Here, in order to further enhance the information capacity of metasurface by simultaneously adjusting multiple light properties, we propose and demonstrate a generalized vortex beam array based on a single metasurface. This metasurface allows for the modulation of various parameters, such as polarization, phase, angular momentum, and 3D diffraction patterns in stereoscopic space. The multidimensional vortex array displays diverse OAM distributions with different intensity profiles and polarization states among different diffraction orders. This array is created using a Dammann vortex metasurface (DVM), which involves the introduction of distinct phase gradients but uniform energy distribution in different vortex elements positioned in XYZ space. Furthermore, by modulating the relative phase between two orthogonal linear polarization channels using a birefringent metasurface with customized rectangular cross-sections, the vectorial characteristics of each diffraction order can be further manipulated, offering additional flexibility for modulation. This approach enables an accurate description of the profile of each vortex element. The presented method significantly increases the information capacity by exploring the 3D optical channels and may find applications in optical communication, optical encryption, and structural beam manipulation.

## Results

**Fig. 1:**
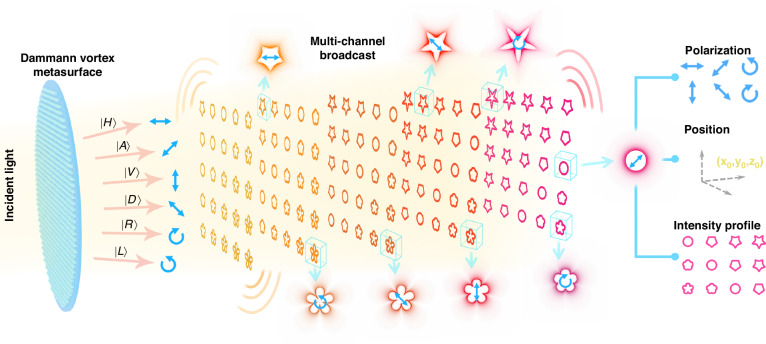
The illustration of dielectric metasurface with vectorial GVB array in 3D space. The incident beams can be tailored either to be circular polarization or linear polarization according to the encoding principle, while the output 3D GVB arrays are characterized by spatially variant vectorial intensity features. In the observation space, the GVB arrays with 5 × 5 × 5 configuration display distinct profiles and states of polarization

As shown in Fig. 1, the full-parameter-modulated GVBs generated by the DVM are arranged in a 3D array and exhibit inhomogeneous intensity profiles. When linearly polarized beams illuminate the metasurface, distinct beam patterns are produced in different diffraction orders in 3D space, each with unique intensity profiles and diverse polarization characteristics. The design principle involves explicitly calculating the phase modulation of the DVM by combining the phase profiles of the vortex array metasurface, Dammann zone plate, and lens factor, while maintaining the relationship between phase delay and amplitude ratio in orthogonal polarization bases. The resulting transmission coefficient is determined by aggregating all diffraction orders based on Taylor expansion theory. Consequently, the output light conveys multiple polarization information, corresponding to various diffraction orders in 3D space, offering additional encryption possibilities.

### Principle

For the control of polarization feature, the transmission coefficient of the DVM with birefringent nature in orthogonal polarization basis can be expressed as follows:

1 $${T}_{{DVM}}=\exp \left(i{\varphi }_{0}\right)\mathop{\sum }\limits_{m=-\infty }^{+\infty }\mathop{\sum }\limits_{n=-\infty }^{+\infty }\mathop{\sum }\limits_{q=-\infty }^{+\infty }\left(\begin{array}{c}{C}_{{mn},x}\cdot {C}_{q,x}\\ {C}_{{mn},y}\cdot {C}_{q,y}\end{array}\right)\exp \left(i\left[\frac{2\pi {mx}}{{\Lambda }_{x}}+\frac{2\pi {ny}}{{\Lambda }_{y}}+\frac{{kq}{r}^{2}}{2{f}_{g}}+m{\varphi }_{x}+n{\varphi }_{y}+q{\varphi }_{z}\right]\right)\left(\begin{array}{c}{{\boldsymbol{e}}}_{{\boldsymbol{x}}}\\ {{\boldsymbol{e}}}_{{\boldsymbol{y}}}\end{array}\right)$$

where ${\varphi }_{0}\left(\theta \right)$, ${\varphi }_{x}\left(\theta \right)$, ${\varphi }_{y}\left(\theta \right)$ and ${\varphi }_{z}\left(\theta \right)$ represent the preset phase feature of GVB (function as four independent design freedoms) in each diffraction order, with ${\varphi }_{(m,n,q)}={\varphi }_{0}+m{\varphi }_{x}+n{\varphi }_{y}+q{\varphi }_{z}$, where ${\varphi }_{0}$ affects the phase features of all diffraction orders, and ${\varphi }_{x}$, ${\varphi }_{y}$, ${\varphi }_{z}$ affects the phase transformation in *x*, *y*, *z* axial. *θ* represents the azimuthal angle of the metasurface plane. ${{\boldsymbol{e}}}_{{\boldsymbol{x}}}$ and ${{\boldsymbol{e}}}_{{\boldsymbol{y}}}$ represent the unit vector in *x* and *y* directions, respectively. ${C}_{{mn}}$ and ${C}_{q}$ represent the transmission coefficient for each diffraction order in transverse planes and along *z* direction, respectively. ${\Lambda }_{x}$ and ${\Lambda }_{y}$ are the period of supercell in *x* and *y* directions, setting as ${\Lambda }_{x}={{Num}}_{x}\cdot {pix}$ and ${\Lambda }_{y}={{Num}}_{y}\cdot {pix}$, where ${{Num}}_{x}$ and ${{Num}}_{y}$ represent the number of lattices within one supercell in *x* and *y* directions based on Dammann optimization principle. *pix* is the lattice constant. $k=2\pi /\lambda$ is wave number and *λ* is the wavelength. $1/{f}_{g}$ decides the focal length interval to obtain an equal distance between adjacent transverse arrays along the *z* direction. The focal position of each plane can be calculated by the Gaussian formula ${f}_{q}={f}_{0}-q\,{{f}_{0}}^{2}/{f}_{g}$, where ${f}_{0}$ is the focal length of the Fourier lens adopted in the experimental system. The diffraction orders $(m,n,q)$ in 3D space provide multiple channels to increase the information capacity. Through carefully tailoring the four phase features ${\varphi }_{0}$, ${\varphi }_{x}$, ${\varphi }_{y}$ and ${\varphi }_{z}$, and integrating the phase modulation and vectorial feature to each diffraction order, the spatial distribution can exhibit abundant functionalities for each diffraction order.

**Fig. 2:**
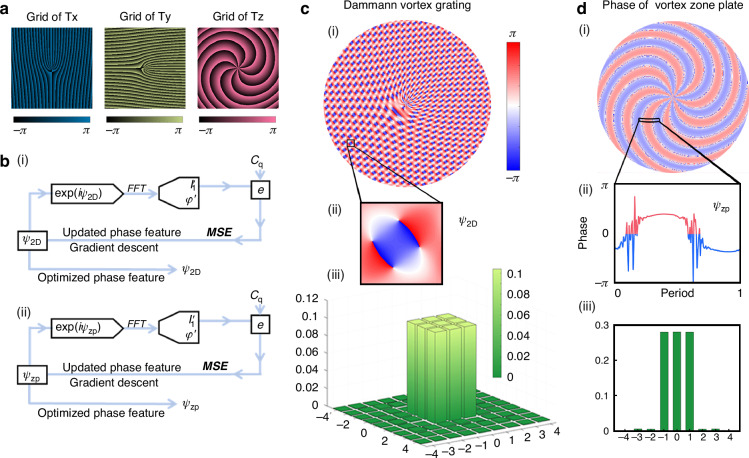
Designing principle of Dammann vortex metasurface. **a** The Fork distribution of horizontal and vertical vortex grating together with the spiral distribution of vortex zone plate. **b** Optimizing the progress of planar diffraction array and Dammann vortex zone plate. **c** Phase feature and diffraction orders of planar diffraction array optimization with 3 × 3 target energy distribution. **d** Filled vortex zone plate phase with Dammann zone plate period

Meanwhile, we propose and demonstrate the GVB by tailoring the azimuthal phase differential distribution, thus the intensity profiles can be altered arbitrarily in our previous works^42,43^. Here, we further expand the capability from two-dimensional modulation to 3D modulation through comprehensive transformation based on Eq. (1). We first form the phase distribution in both *x* and *y* directions by setting ${T}_{x}={e}^{i\left(\frac{2\pi x}{{\Lambda }_{x}}+{\varphi }_{x}\right)}$ and ${T}_{y}={e}^{i(\frac{2\pi y}{{\Lambda }_{y}}+{\varphi }_{y})}$ with characteristic fork-like distribution, as shown in Fig. 2a. The Dammann spiral zone plate possesses helical distribution and can generate different GVBs along longitudinal direction, as shown in Fig. 2a. The number of spiral lobes around the center corresponds to the topological charge number of vortex beam in *z* direction, and the phase information of the spiral zone plate can be expressed as ${T}_{z}={e}^{i(\frac{{\rm{k}}{r}^{2}}{2{f}_{g}}+{\varphi }_{z})}$. By jointly optimizing the transmission coefficients of ${T}_{x}$, ${T}_{y}$, and ${T}_{z}$, the desired energy distribution can be realized in each diffraction order, with the optimization flow charts shown in Fig. 2b. ${\psi }_{2D}$, ${\psi }_{{zp}}$ represent the optimized phase information of planar diffraction arrays and Dammann zone plate.

Through Dammann optimization, we acquire the optimized phase profile within the supercell (composed of several lattices), which can later be incorporated to the above analytical phase profiles (${\varphi }_{x}$, ${\varphi }_{y}$ and ${\varphi }_{z}$) for uniform energy distribution, as well as ensuring large numerical aperture for displaying enough number of diffraction orders, as shown in Fig. 2c(i, ii) and d(i, ii). We design 3 × 3 arrays in the transverse plane with *m* = −1,0,1 and *n* = −1,0,1, for the demonstration. Meanwhile, all other diffraction orders have negligible energy because of such optimization. These chosen nine diffraction orders have a total energy proportion $\sum {I}_{{mn}}$ over 95%, with the energy fluctuation ${I}_{{mn}}$ among the diffraction orders less than 0.1%, as shown in Fig. 2c(iii). The energy proportion corresponds to ${I}_{{mn}}={\left|{C}_{{mn}}\right|}^{2}$, in which ${C}_{{mn}}$ is the optimized transmission coefficient of each diffraction order. For a simplified model without vectorial effect, the target diffraction orders have the same values with ${C}_{{mn},x}={C}_{{mn},y}$ and ${C}_{q,x}={C}_{q,y}$ for two orthogonal polarization channels. Similarly, in the propagating direction, the target diffraction orders are chosen as *q* = −1, 0, 1 to form the final 3D arrays by integrating the optimization phase profile of the Dammann zone plate, as shown in Fig. 2d(i, ii). The diffraction energy distribution of Dammann zone plates is shown in Fig. 2d(iii), where the overall energy proportion $\sum {I}_{q}$ of target orders is over 85%, and the energy fluctuation ${I}_{q}$ of each order is less than 1.3%.

### Verification with geometric metasurface

**Fig. 3:**
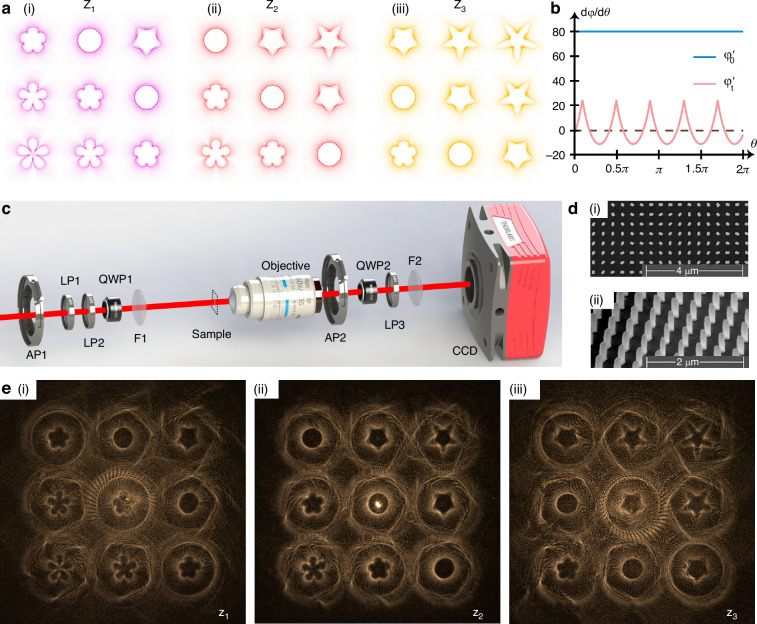
Design and experimental verification of geometric metasurface. **a** The pattern types of the special GVB array. **b** The phase differential distribution of specific features. **c** Optical setup for generating 3D GVB array with fabricated metasurface. AP aperture, LP linear polarizer, QWP quarter-wave plate, F focus lens, CCD charge-coupled device. **d** Top view and side view of SEM images of one fabricated silicon metasurface sample. The metasurface holograms are composed of 800 × 800 nanofins with the same size but different orientation angles. **e** Experimental results of different observation planes located at Z_1_, Z_2_ and Z_3_

In order to verify the generation of stereoscopic GVB arrays with DVM, here we design the 3 × 3 × 3 vortex array with 27 different orders in 3D space for demonstration. Such DVM is composed of dielectric silicon pillar antennas fabricated by electron beam lithography and reactive Ion etching. For a uniform polarization feature of all diffraction orders, the scalar DVM is designed based on the geometric phase principle with the same geometric size but different rotational angles. As shown in Fig. 3a, the GVB array of 27 orders is defined with different phase gradient (${\varphi}_{x},{\varphi}_{y},{\varphi}_{z}$) information. The complete information of a specific diffraction order can be expressed as phase features of ${\varphi}_{m,n,q}={\varphi}_{0}+m{\varphi} _{x}+n{\varphi}_{y}+q{\varphi}_{z}$. Based on the connection between beam intensity and phase information of GVB, the radius ${R}_{m,n,q}\left(\theta \right)$ in k space is proportional to the phase gradient as ${R}_{m,n,q}\left(\theta \right)\propto \frac{d{\varphi }_{m,n,q}\left(\theta \right)}{d\theta }$, so that the beam shape of each diffraction order $({\rm{m}},{\rm{n}},{\rm{q}})$ follows the rule of ${R}_{m,n,q}\left(\theta \right)\propto m\frac{d{\varphi }_{x}\left(\theta \right)}{d\theta}+n\frac{d{\varphi}_{y}\left(\theta \right)}{d\theta}+q\frac{d{\varphi}_{z}\left(\theta \right)}{d\theta }+\frac{d{\varphi}_{0}\left(\theta \right)}{d\theta}$. In Fig. 3b, ${\varphi }_{0}$ is defined as ordinary vortex beams with constant phase diffraction ${{\varphi }^{{\prime} }}_{0}=80$. Meanwhile, the other three modulated phase elements are defined as the same feature ${\varphi }_{t}$ (${\varphi }_{x}={\varphi }_{y}={\varphi }_{z}={\varphi }_{t}$) with ${\varphi {\prime} }_{t}=f\left({\rm{\theta }}\right)/2-40$ and five-fold rotational symmetry (C5). $f\left({\rm{\theta}}\right)={\sum}_{i=1}^{5}\frac{1}{\left|\sin {\rm{}} \theta -\tan {\rm{}}\frac{\pi }{5}(i\pm 0.5)\cos {\rm{}}\theta \right|}$ defines the five-star curve. That is, for each diffraction order, the GVB profile can be expressed as: ${R}_{m,n,q}({\rm{\theta}})={R}_{0}={R}_{0}+\left(m+n+q\right){R}_{t}$, where *R*_*t*_ is proportional to ${\varphi }_{t}$. Hence, for these orders that have the same value of $m+n+q$, they have the same intensity profile. Specifically, for the diffraction orders with $m+n+q=0$, the GVB patterns are reduced to a doughnut shape.

In order to verify the effect of DVM, the geometric metasurface is chosen due to fabrication robustness and broadband operation superiority. The diameter of the metasurface is 480 μm, and the height of 600 nm. As shown in Fig. S1, the length, width and height of each silicon antenna are selected to exhibit high polarization conversion for implementing geometry phase (Details can be found in Supplementary Information Section D). The geometric phase is related to the azimuthal angle of anisotropic nanofins obeying $\varphi =2\theta$. Hence, DVM composed of silicon antenna arrays with different azimuthal angles can implement the phase profile faithfully. For the lens factor design, the focal length of the Fourier lens is chosen as ${f}_{0}$ = 200 mm, and the focus modulation parameter is chosen as ${f}_{g}$ = 800 mm. Therefore, the observation planes are located at ${f}_{-1}$ = 150 mm, ${f}_{0}$ = 200 mm and ${f}_{1}$ = 250 mm, respectively. The number of lattices within the supercell is ${{Num}}_{x}={{Num}}_{y}$ = 8, and the Numerical Aperture (NA) of the full view space is ${NA}$ = 0.707 with the wavelength of 800 nm and the antenna period of 400 nm.

The experimental setup and the scanning electron microscope (SEM) images of the fabricated DVM are shown in Fig. 3c and d, respectively. Note that the intensity profile of each diffraction order in the 3D array of GVB is spatially variant, while the intensity profiles are the same for these orders with the same summation of $m+n+q$. In each observation plane, besides the target intensity distribution, there is also some crosstalk from the angular spectrum distributions of the other two observation planes, as shown in Fig. 3e. The geometric metasurfaces usually possess a broadband working spectrum. In addition to the sample #1, another two samples with different designs of ${\varphi }_{x}\,\ne\, {\varphi }_{y}\,\ne\, {\varphi }_{z}$ are fabricated for various GVB arrays in 3D space as shown in Fig. S2 (Details of sample #2 and sample #3 are described in Supplementary Information Section B). In the experiment, we measure the broadband effect of all three samples with different wavelengths ranging from 700 nm to 900 nm, as shown in Figs. S3–S5. The spatially variant distributions and even the mathematical representations can be obtained therein.

### Vectorial 3D GVB array

**Fig. 4:**
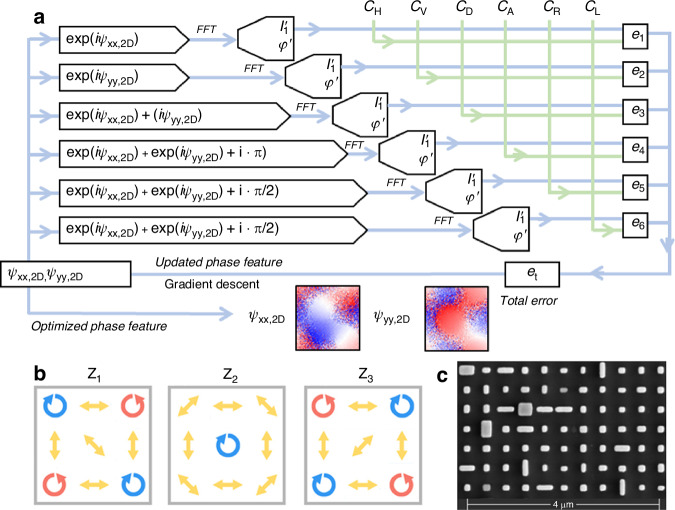
Sketch of generating GVVB based on vector Dammann metasurface. **a** Optimizing progress of vector Dammann grating and vector Dammann zone plate. **b** Diffraction distribution of 3 × 3 × 3 orders at typical polarization states. **c** Top view of scanning electron microscopy images of one fabricated silicon resonate metasurface sample

Apart from the GVB array with scalar feature, we further introduce polarization modulation with different orthogonal basis to form the 3D GVB arrays by using DVM. Here, we choose a birefringent metasurface that can modulate the phase profiles independently for orthogonal polarization channels by tailoring the rectangular cross-section of nanofins, as shown in Fig. S6. We optimize the phase features for the *x*- and *y*-polarization simultaneously, and the energy distribution and the desired polarization state of the target diffraction order are determined by optimization of six special polarization states. During the optimization, there are two kinds of optimizing variables ${\psi }_{{xx},2D}$ and ${\psi }_{{yy},2D}$, and six kinds of constraint conditions of ${\left|{C}_{H}\right|}^{2}$, ${\left|{C}_{V}\right|}^{2}$, ${\left|{C}_{D}\right|}^{2}$, ${\left|{C}_{A}\right|}^{2}$, ${\left|{C}_{R}\right|}^{2}$ and ${\left|{C}_{L}\right|}^{2}$, where ${C}_{H}$ and ${C}_{V}$ represent the coefficient matrix of ${C}_{{mn},x}$ and ${C}_{{mn},y}$ in Eq. (1) to form vectorial Dammann optimization, as shown in Fig. 4a. Similarly, for vectorial Dammann zone plate, ${C}_{H}$ and ${C}_{V}$ represent the coefficient matrix of ${C}_{q,x}$ and ${C}_{q,y}$ for each diffraction orders along propagation direction. ${C}_{D}$, ${C}_{A}$, ${C}_{R}$ and ${C}_{L}$ correspond to $({C}_{H}+{C}_{V})/\sqrt{2}$, $({C}_{H}-{C}_{V})/\sqrt{2}$, $({C}_{H}+i{C}_{V})/\sqrt{2}$ and $({C}_{H}-i{C}_{V})/\sqrt{2}$, respectively. This kind of joint optimization method not only defines corresponding energy distribution for each special polarization states, but also can control the phase difference between the *x-* and *y*-polarization channels with ${|\cos ({\psi }_{{xx}}-{\psi }_{{yy}})|}^{2}$ and ${|\sin ({\psi}_{{xx}}-{\psi }_{{yy}})|}^{2}$.${|\sin ({\psi }_{{xx}}-{\psi }_{{yy}})|}^{2}$. Those constraints of energy distribution and phase difference between the *x*- and *y*-polarization channels guarantee the vectorial Dammann optimization in full polarization space. Similarly, the vectorial Dammann zone plate also obtains special distributions of amplitude and polarization states at different diffraction orders through the combination of the *x*- and *y*-polarization channels. Based on the above method, the 3D vectorial DVM can be obtained by combining the phase information of the planar vortex array and the Dammann vortex zone plate in orthogonal polarization channels, and the comprehensive polarization features of 3 × 3 × 3 orders are combined with *xy* planar and *z* direction modulation.

Based on dielectric metasurface with resonance phase feature, we design two vectorial DVM samples (sample #4 and sample #5) to generate full-parameter-modulated 3D GVB arrays. The vectorial optimization results are shown in Figs. S7 and S8. Based on Eq. (1), the 3D vectorial DVM can be fabricated with the phase composition of a vectorial planar diffraction array and a vectorial Dammann zone plate. We design 3 × 3 × 3 vectorial GVB arrays for demonstration. The polarization states of diffraction orders in each observation plane are shown in Fig. 4b for sample #4 and Fig. S9 for sample #5. The vectorial DVM adopts a birefringent metasurface for vectorial modulation, which is composed of different nano pillars with rectangular cross-section determined by the independent phase modulation in the *x*- and *y*-polarization channels. The antennas are designed and fabricated with a height of 600 nm, period of 400 nm, and the length and width ranging from 70 nm to 300 nm, as shown in Fig. S6. The diameter of the metasurface is 480 μm diameter and the working wavelength is 800 nm. The length and width sizes of antennas are chosen based on the phase combinations of (*ψ*_*xx*_, *ψ*_*yy*_). Figure 4c shows the SEM of vectorial DVM, and more details can be found in Supplementary Information Section C.

Note that all the 27 diffractions orders of sample #4 have the same intensity profiles of sample #1 but various polarization states, covering six polarization states as follows: vertical polarization (V), horizontal polarization (H), Anti-diagonal polarization (135°, A), diagonal polarization (45°, D), left-handedness circular polarization (L), right-handedness circular polarization (R). In order to realize the special vector feature of 27 diffraction orders, two steps are adopted for optimization. First, the vectorial Dammann vortex metasurface is designed with *q* = 0 to generate 3 × 3 diffraction orders in *xy* plane, which possesses equal energy distribution but varying vector feature in each order. Then, for the other two planes along the *z* direction (with *q* = −1 and *q* = 1), L and R polarization are introduced by considering the integral modulation of each plane. For example, the polarization state of the diffraction order (*m*, *n*, *q*) can be set as follows: E(0,0,0) = $${\left[\begin{array}{cc}1 & {\rm{i}}\end{array}\right]}^{T}$$, E(0,0,-1)$$={\left[\begin{array}{cc}1 & -1\end{array}\right]}^{T}$$ and $${\rm{E}}(0,0,1)={\left[\begin{array}{cc}1 & 1\end{array}\right]}^{T}$$, which represents the circular polarization in the Z_2_ plane, and the diagonal and anti-diagonal linear polarization in the Z_1_ and Z_3_ planes, respectively. The polarization features of all other diffraction orders can also be analyzed with the same methods.

**Fig. 5:**
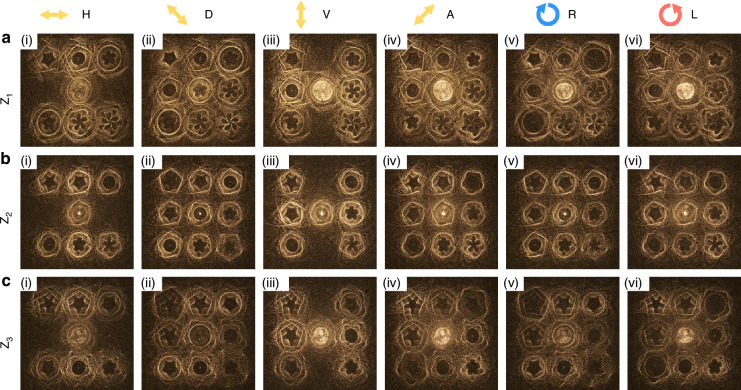
Experimental results of vectorial GVB array in 3D space. Figure 5**a**–**c** correspond to different observation depths Z_1_, Z_2_ and Z_3_. The observed GVB patterns are consistent with target shapes and polarization. Under six typical polarization states, the diffraction orders with different polarization state appear and vanish alternately, in line with the designed polarization state

The experimental results of such vectorial 3D GVB arrays of sample #4 are shown in Fig. 5. We modulate the output polarizations to observe the vectorial feature based on Malus’ principle through the detection of energy distribution within each diffraction order. That is, we choose $|H\rangle ,|V\rangle ,{|D}\rangle ,|A\rangle ,|L\rangle ,|R\rangle$ by using the combination of a polarization analyzer and a quarter-wave plate to obtain the output results. Note that the diffraction orders with the varying polarization states can be alternatively annihilated when the analyzer is orthogonal to their polarization states. Each order possesses distinct polarizations and intensity profiles at different observation planes in space. Compared to the geometric metasurface (sample #1) with scalar distributions, the birefringent metasurface (sample #4) has a relatively strong central zero point, which is focused and defocused at different planes.

In order to verify the arbitrary polarization manipulation of such a vectorial DVM, we design another sample (sample #5) that possesses elliptical polarization feature. Different phases are preset (${\varphi }_{x}\,\ne\, {\varphi }_{y}\,\ne\, {\varphi }_{z}$) to introduce abundant intensity features in each diffraction order. Meanwhile, the polarization states in each plane are also designed with nine different polarization information, as shown in Fig. S9. In the vectorial Dammann optimization process, the different diffraction order polarization states of the Z_2_ plane can be optimized simultaneously, and the vectorial changes of different depths can be controlled by optimizing the vector Dammann zone plate.

Similarly, in addition to the experimental measurement under the working wavelength of 800 nm, we also test the broadband effect of the resonance metasurface with sample #5 at Z_2_ plane, as shown in Fig. S9, with the wavelength ranging from 680 nm to 900 nm. The broadband transmission efficiency of such metasurface is shown in Fig. S10 (Details can be found in Supplementary Information Section D).

## Discussion

In summary, we have proposed and demonstrated the generation of a 3D GVB array with controllable amplitude, phase, polarization, and diffraction orders using a novel kind of DVM. To the best of our knowledge, we achieve for the first time the vectorial diffraction arrays with arbitrary beam patterns by employing vectorial DVM. Such a metasurface design method fully utilizes the multidimensional information of the wavefront through joint optimization control of phase profiles and based on the Taylor expansion method. Such a method can be used for optical wireless broadcasting in 3D locations, with large capacity, customized information passing with different vectorial and intensity distributions. It can also be applied to various applications such as optical trapping, optical computing, optical communication, etc.

## Materials and methods

The experimental setup is shown in Fig. 3c. The circularly polarized beam can be obtained by using the linear polarizers (LP1 and LP2) and quarter-wave plate (QWP1), and illuminates the metasurface. The objective (60×, NA = 0.85) is utilized to collect the output light of the metasurface for easy capture by the camera. In the experiment, different planes of the GVB array can be observed by moving the detection system back and forth. Such a setup is also applicable for the test of vectorial DVM, by adjusting the incident beam to 45° linear polarization. The vectorial GVB arrays can be analyzed by dynamically rotating the polarizers (QWP2 and LP3) to observe different polarization states based on Malus’ principle.

## Supplementary information

Supplementary Information for Full-parameter-modulated three-dimensional vectorial generalized vortex array

## Data Availability

The data that support the findings of this study are available from the corresponding author upon reasonable request.
