# Supplementary material for: Full-parameter-modulated three-dimensional vectorial generalized vortex array
Source: Light Sci Appl. 2026 Jan 1;15:7. doi: 10.1038/s41377-025-02065-9 (PMC12756242; doi:10.1038/s41377-025-02065-9)
Supplement: Supplementary file 1 — Supplementary Information for Full-parameter-modulated three-dimensional vectorial generalized vortex array [file 41377_2025_2065_MOESM1_ESM.docx]

# **Supplementary Information**

**Full-parameter-modulated three-dimensional vectorial generalized vortex array**

*Xue Zhang^1,2^, Yang Cui^1^, Yanjie Chen^1^, Xiaowei Li^3^, Junjie Li^4^, Wenqiao Shi^5^, Jian Chen^6,5^, Zhaogang Dong^7,8^, Yongtian Wang^1^, Cheng-Wei Qiu^5,^ ^[[1]](#footnote-1)^, Shuang Zhang^9,^ ^[[2]](#footnote-2)^ and Lingling Huang^1,^ ^[[3]](#footnote-3)^*

^1^ Beijing Engineering Research Center of Mixed Reality and Advanced Display, School of Optics and Photonics, Beijing Institute of Technology, Beijing, 100081, China

^2^Iinstitute of Photonic chips, University of Shanghai for Science and technology, Shanghai 200093, China.

^3^  Laser Micro/Nano-Fabrication Laboratory, School of Mechanical Engineering, Beijing Institute of Technology, Beijing 100081, China

^4^ Beijing National Laboratory for Condensed Matter Physics, Institute of Physics, Chinese Academy of Sciences, Beijing 100191, China

^5^ Department of Electrical & Computer Engineering, National University of Singapore, Kent Ridge, Singapore 117583

^6^ School of Optical-Electrical and Computer Engineering, University of Shanghai for Science and Technology, Shanghai 200093, China

^7^ Science, Mathematics, and Technology (SMT), Singapore University of Technology and

Design (SUTD), 8 Somapah Road, Singapore 487372

^8^ Quantum Innovation Centre (Q.InC), Agency for Science Technology and Research (A*STAR), 2 Fusionopolis Way, Innovis #08-03, Singapore 138634, Republic of Singapore

^9^ Department of Physics, University of Hong Kong, Hong Kong, China

## **Antennas selection of geometric metasurface**

The scalar DVM is designed with dielectric geometric phase antennas, and the phase information is depicted in Fig. S1(a). The silicon dielectric antenna adopts a rectangular nanofin structure of the same size, with different rotation angles corresponding to half of the phase shift. The antenna distribution diagrams are shown in Fig. S1(b,c). For a circularly polarized incident beam, the geometric metasurface provides target phase modulation in orthogonal circular polarization. To meet the special fabrication demands at the working wavelength of 800 nm, with remaining the period of 400 nm and the height of 600 nm, we scanned the antenna sizes from 70 nm to 300 nm in length and width. The circular transmission distribution and phase difference are shown in Fig. S1(d,e). The selected size of 220 nm×130 nm achieves the highest circular transmission above 90%, with a phase difference of π/2, as marked in Fig. S1(d,e). The phase modulation characteristics of the antenna by rotating the azimuthal angle satisfy the geometric phase principle, as illustrated in Fig. S1(f)..

## **Stereoscopic GVB arrays generation based on DVM**

Compared with Sample #1 in Fig. 3，by setting different$\varphi_{x}$, $\varphi_{y}$ and $\varphi_{z}$ , the stereoscopic diffraction orders will present 27 kinds of different GVB patterns. Here, we design another two Sample #2 and #3 with GVB arrays of the quadrilateral distributed features, as shown in Fig. S2. For Sample #2, $R_{0,0,0}$, $R_{0,0,1}$,$R_{1,0,0}$, and $R_{0,1,0}$ correspond to different GVB shapes of circle, square, four-corner star and its counterpart with 45° rotation, respectively. Since the variable radius $R_{x}$，$R_{y}$ and $R_{z}$ are all different from each other, total 27 different GVB shapes can be generated in three different planes by Sample #2. For Sample #3, ${\varphi'}_{x}$，${\varphi'}_{y}$ and ${\varphi'}_{z}$ are special mathematical functions as ${\varphi'}_{0}=80$，${\varphi'}_{x}=8sin(6\theta)$，${\varphi'}_{y}=12sin(6\theta)$， and ${\varphi'}_{z}=8sin(3\theta+\pi/4)$. Therefore, the stereoscopic GVB array can perform mathematical functions with $\varphi_{m,n,q}=80\theta-4/3m\cos(6\theta)-2n\cos(6\theta)-8/3q\cos(3\theta+\pi/4)$*,* and spatial functions can also be expressed by following the rule of$R_{m,n,q}\left( \theta\right)\propto m\frac{d\varphi_{x}\left( \theta\right)}{d\theta}+n\frac{d\varphi_{y}\left( \theta\right)}{d\theta}+q\frac{d\varphi_{z}\left( \theta\right)}{d\theta}+\frac{d\varphi_{0}\left( \theta\right)}{d\theta}$. Compared with 2D GVB arrays, there is an extra adjustable parameter that can be taken into computation, with increased information capacity. In the experiment, we test the broadband performance of three different samples with several working wavelengths ranging from 720 nm to 900 nm. The experimental results are shown from Fig. S3 to Fig. S5.

## **C. Antennas selection with birefringent metasurface**

For the antenna selection of birefringent metasurfaces, the basic parameters of rectangular nanofins are as follows: height of 600 nm, pixel period of 400 nm, and the length and width ranging from 70 nm to 300 nm. The distribution of antenna array of the vectorial DVM is shown in Fig. S6(a,b), with different sizes of rectangular nano pillars. The transmission amplitude and phase are varied by modulating the rectangular cross sections, as depicted in Fig. S6(c,d). The two phases *ψ_xx_* and *ψ_yy_* in the two independent polarization channels of such birefringent metasurface can cover −*π* to *π* (Fig. S6e, 6f), which can ensure the arbitrary combination to achieve polarization control of each diffraction orders.

## **D. 3D GVB arrays with spatially variant polarizations**

In addition to Sample #4 mentioned in the main text, we also designed sample #5 for a vectorial DVM corresponding with spatially variant polarization states. The intensity profiles of GVB arrays are the same as Sample #3, containing 27 different profiles in each diffraction order and 14 kinds of different polarization states. The polarization states contain not only six typical polarizations such as |H>, |V>, |D>, |A>, |L>, |R>, but also 4 kinds of elliptical polarization and another 4 kinds of linear polarizations.

As shown in Fig.S7 and Fig.S8, we provide the optimization process for the 3D vectorial GVB arrays by using jointly optimization method. The 2D energy distribution of the target 3×3 diffraction orders in XY planes, including the polarization features, reach up to 85% for sample #4 and 82% for sample #5. For the vectorial zone plate (ZP) optimization, the energy distribution of the target three planes reaches up to 81% for sample #4 and sample #5. Additionally, the energy distribution of the diffraction orders with different polarization is relatively uniform, with deviation rate smaller than 3 × 10^-6^. We also provide six types of polarization analysis under different polarization conditions for 2D plane and zone plate respectively, that is, |H>, |V>, |A>, |D>, |R>, |L>, to analyze the vectorial feature of each order. The simulation results can well support the desired ones.

The experimental results of Sample #4 and Sample #5 are shown in Fig. S9. To test the broadband performance of vectorial DVM, we selected Sample #5 under right-handedness circular polarization (|R>) at Z_2_ plane, as illustrated in Fig. S10. The testing wavelengths range from 680 nm to 960 nm. Note the birefringent metasurface also exhibits satisfactory modulation across such a broad spectrum.

## **E. 3D GVB arrays with 5×5×5 diffraction orders and vectorial GVB arrays**

The full-parameter-modulated 3D vectorial generalized vortex array design method discussed in this article is applicable to flexible diffraction scenarios. As illustrated in Fig. 1, a total of 125 spatially arranged beams of arbitrary shapes can be generated, with corresponding polarization features through a 5×5×5 array. Fig.S11 presents a diagram of the simulated light field of the scalar spatial vortex array corresponding to Fig.1. In Fig.S11 (a), the propagation trajectories of such GVB arrays are depicted, where the five target Z planes are indicated. In each transverse plane, one can observe the 5×5 beam arrays. The phase characteristics $\varphi_{0}$ is defined with constant phase differential of ${\varphi^{'}}_{0}=40$. Meanwhile, the other three modulated phase design freedom are defined with the same feature $\varphi_{t}$ ($\varphi_{x}=\varphi_{y}=\varphi_{z}=\varphi_{t}$) with ${\varphi'}_{t}=f\left( \theta\right)/4-10$, where $f\left( \theta\right)=\sum_{i=1}^{5} \frac{1}{\left| sin\theta-tan\frac{\pi}{5}(i\pm0.5)cos\theta\right|}$ defines the five-star curve and exhibit C5 symmetry. For each diffraction order, the GVB profile can be expressed as: $R_{m,n,q}(\theta)=R_{0}=R_{0}+\left( m+n+q \right)R_{t}$, where *R_t_* is proportional to $\varphi_{t}$ as $R_{t}\left( \theta\right)\propto\frac{d\varphi_{t}\left( \theta\right)}{d\theta}$. Fig.S12 illustrates simulation diagrams of vectorial 5×5×5 GVB arrays, each exhibiting distinct polarization features, showcasing a variety of intensity profiles, and demonstrating the large capacity of such metasurface.

**Figures:**


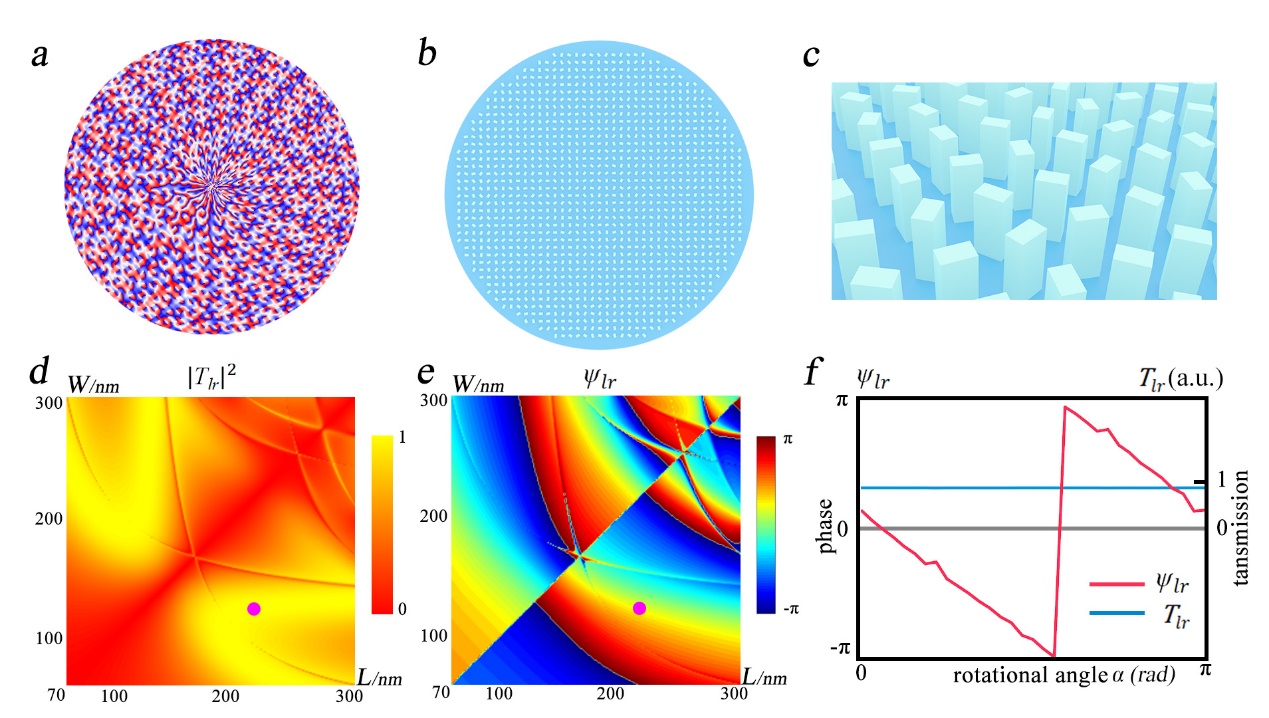


**Fig.S1 Design of geometric phase metasurface.** **a.** The phase information of DVM with geometric phase features**. b.** Antennas distribution of overall metasurface in top view. **c.** Antennas distribution of metasurface in oblique view. **d.** The transmission distribution of swept antennas with $T_{lr}$ feature, and the chosen size is marked with pink point. **e.** The phase of swept antennas with $\varphi_{lr}$ feature, and the chosen size is marked with pink points. **f.** Phase change $\varphi$ and the transmission $\left| T_{lr} \right|$ with opposite circular-polarization with varied orientation angle $\alpha$ from 0 to $\pi$ with 37 steps at wavelength *λ*=800 nm.


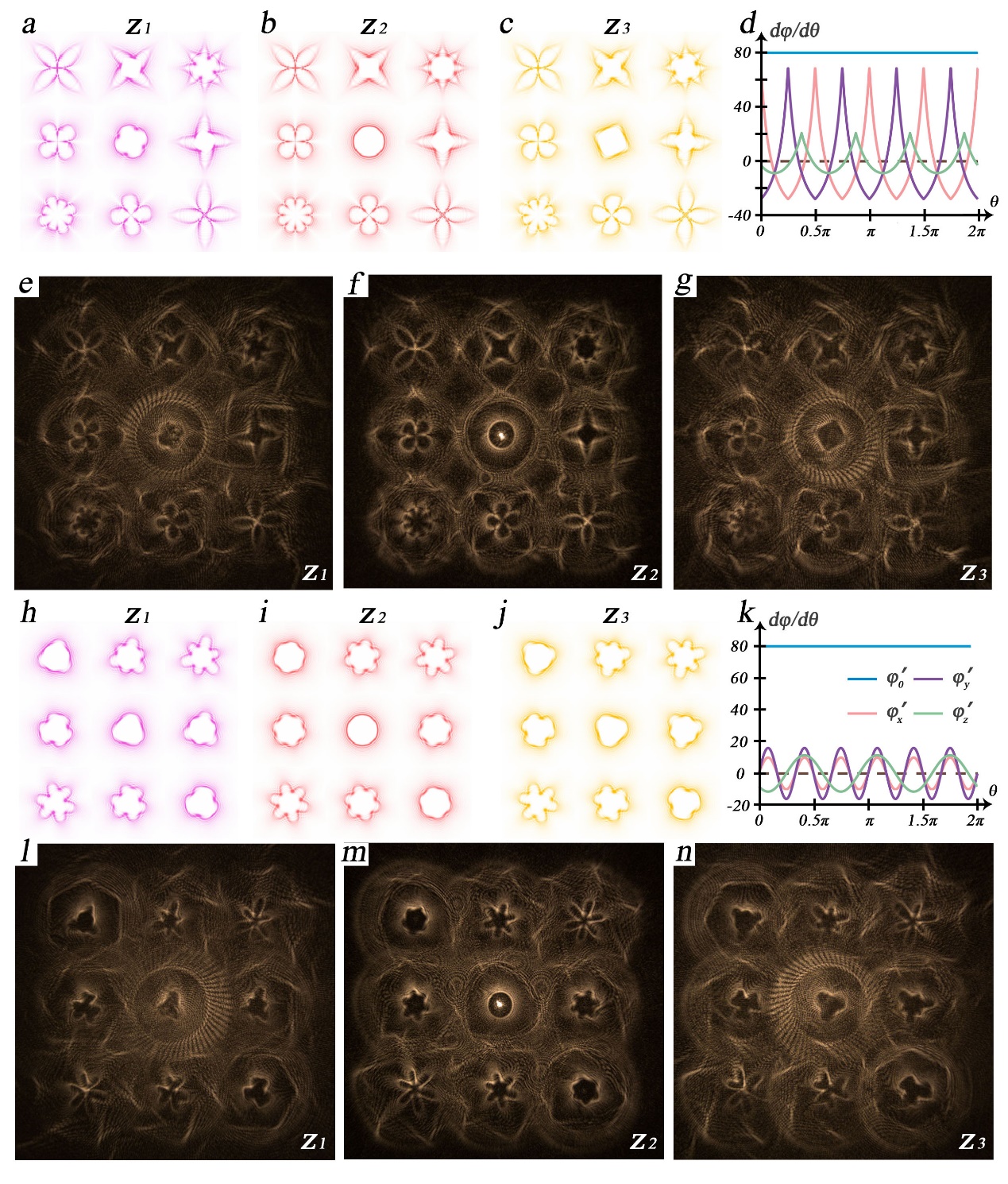


**Fig.S2 GVB intensity profiles with different settings of *φ*_0_, *φ_x_*, *φ_y_* and *φ_z_* of Sample #2 and Sample #3. a-c.** The intensity profiles GVB array in three different planes for Sample #2. **d.** The phase differential distribution of *φ*_0_, *φ_x_*, *φ_y_* and *φ_z_* along the azimuthal direction. **e-g.** Experimental results at different observation planes. **h-j.** The intensity profiles GVB array in three different planes for Sample #3. **k.** The phase differential distribution of *φ*_0_, *φ_x_*, *φ_y_* and *φ_z_* along the azimuthal direction. **l-n.** experimental results of different observation planes.


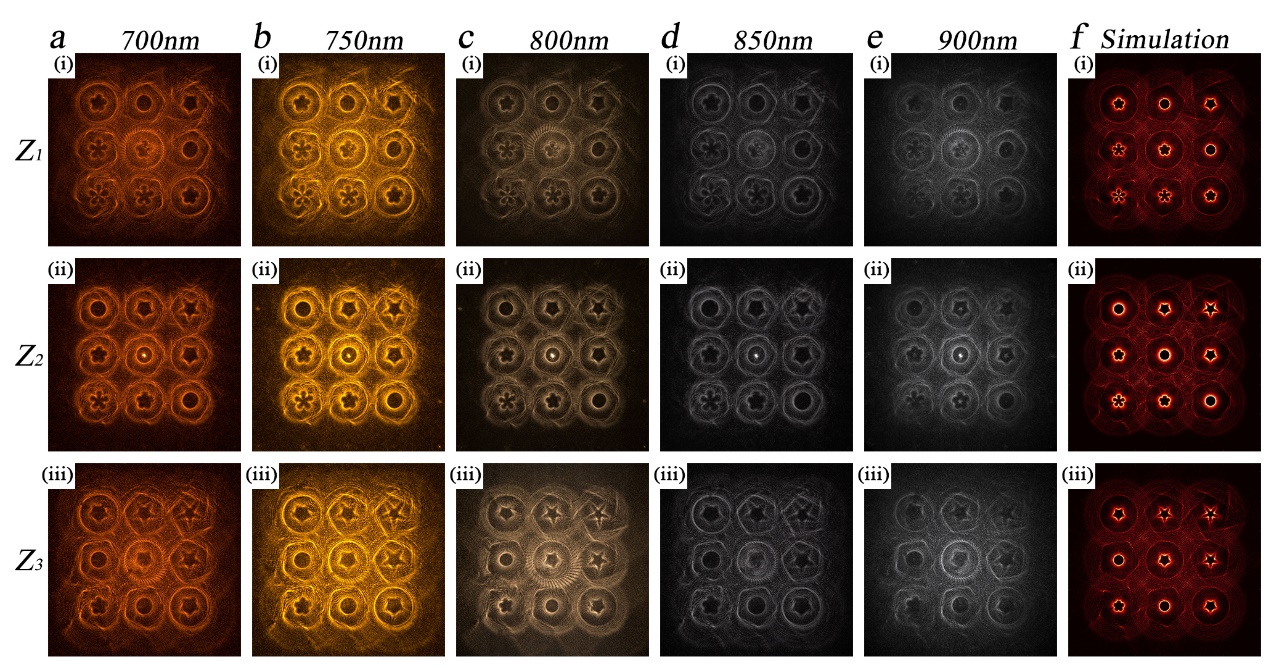


**Fig.S3 Experimental results of broadband verification of DVM at different wavelengths ranging from 700 nm to 900 nm for Sample #1.**


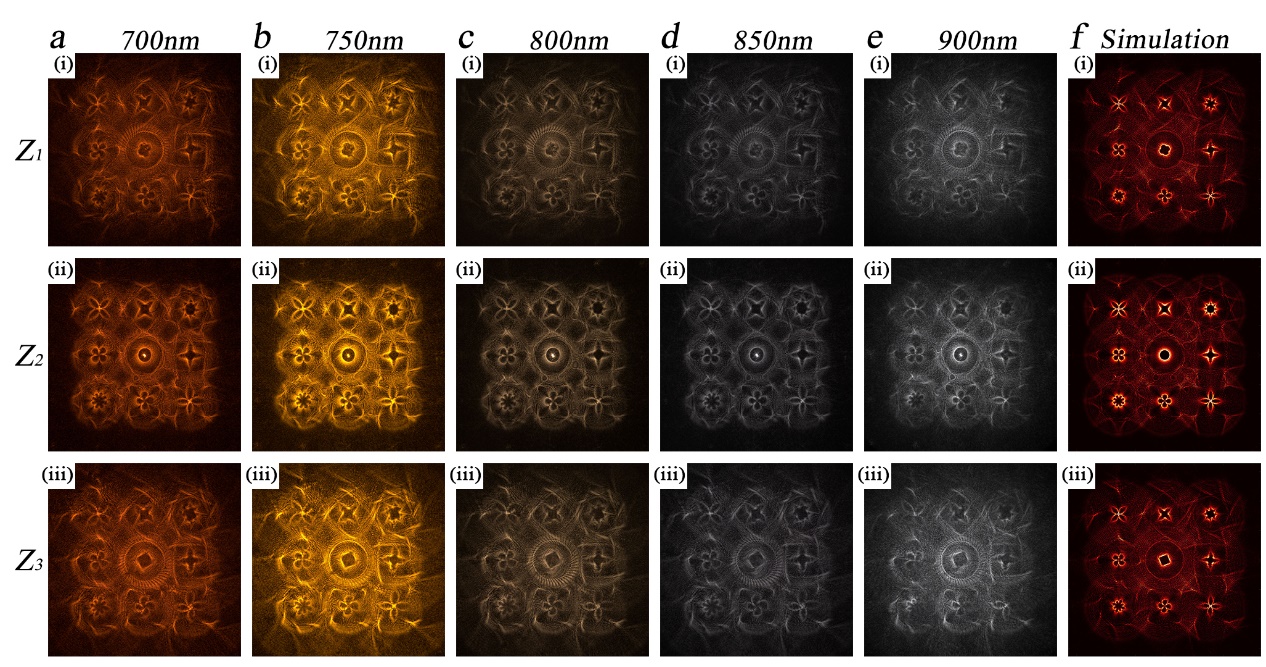


**Fig. S4 Experimental results of broadband verification of DVM at different wavelengths ranging from 700 nm to 900 nm for Sample #2.**


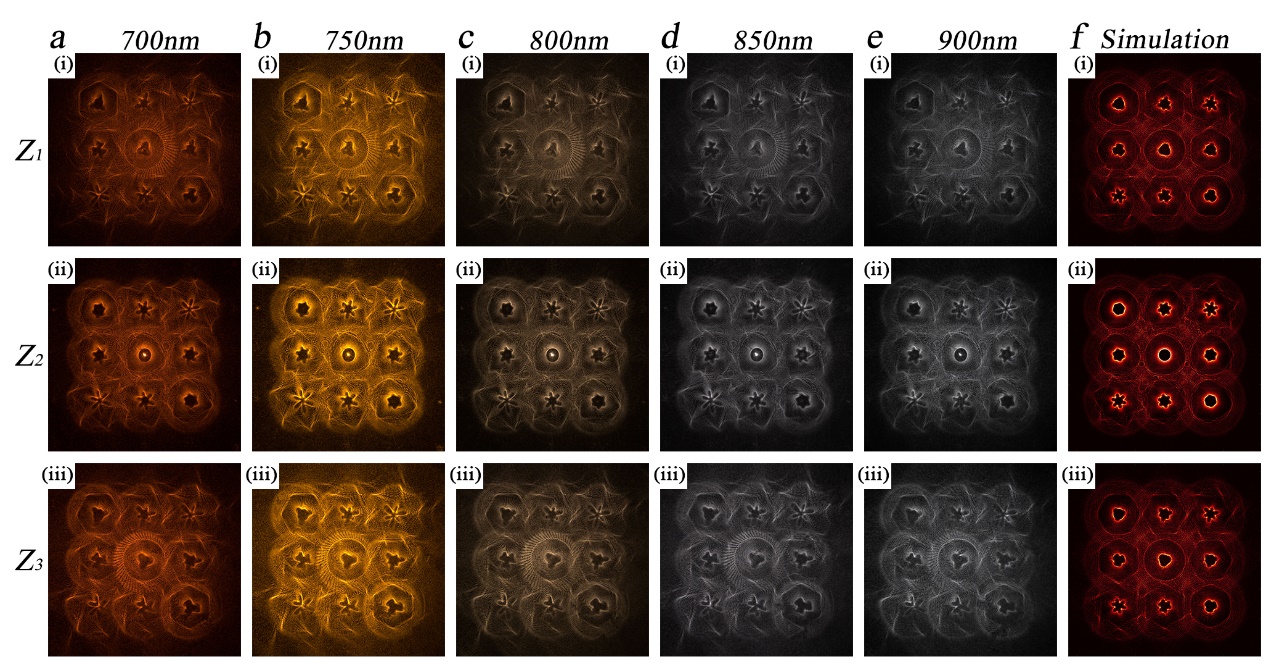


**Fig. S5 Experimental results of broadband verification of DVM at different wavelengths ranging from 700 nm to 900 nm for Sample #3.**


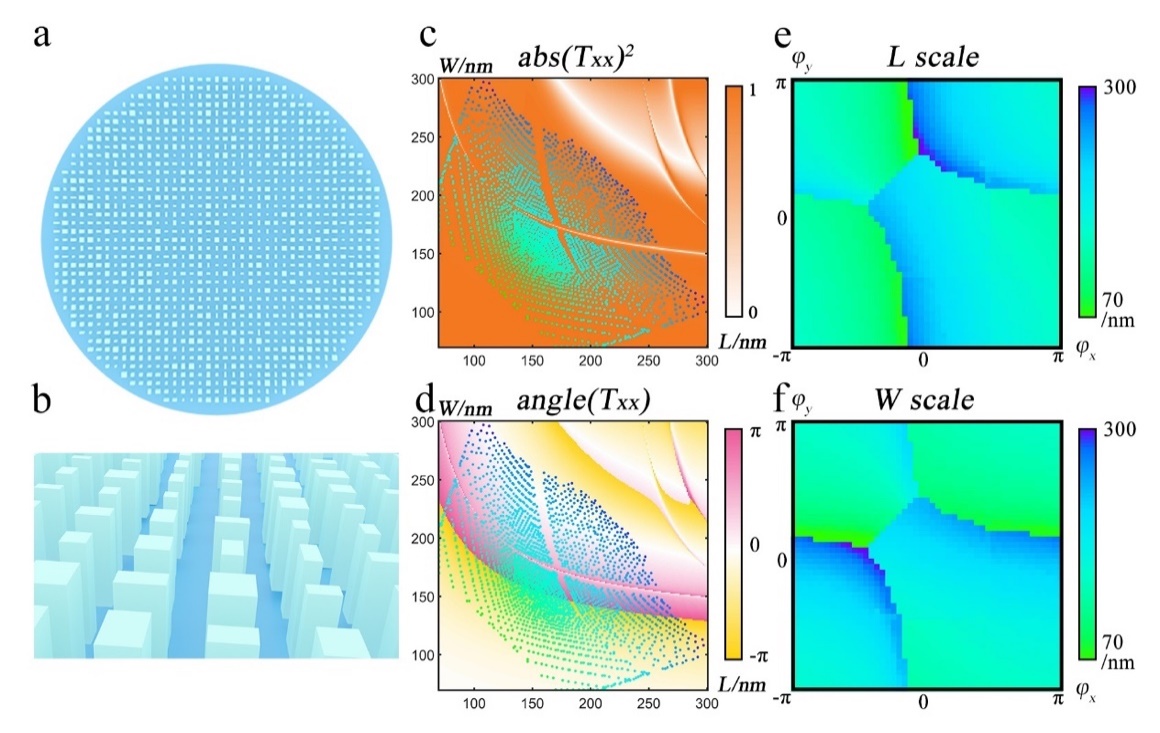


**Fig. S6 Design of birefringent metasurface. a, b.** The geometry design of birefringent metasurface in top and oblique view, respectively. **c-d.** The transmission amplitude and phase for rectangular nano pillars for *x*-*x* input-output polarization. **e, f.** Chosen length and width of nano pillars, whose phase modulation in *x*-*x* and *y*-*y* input-output polarization channels can cover the full 2π ranges.


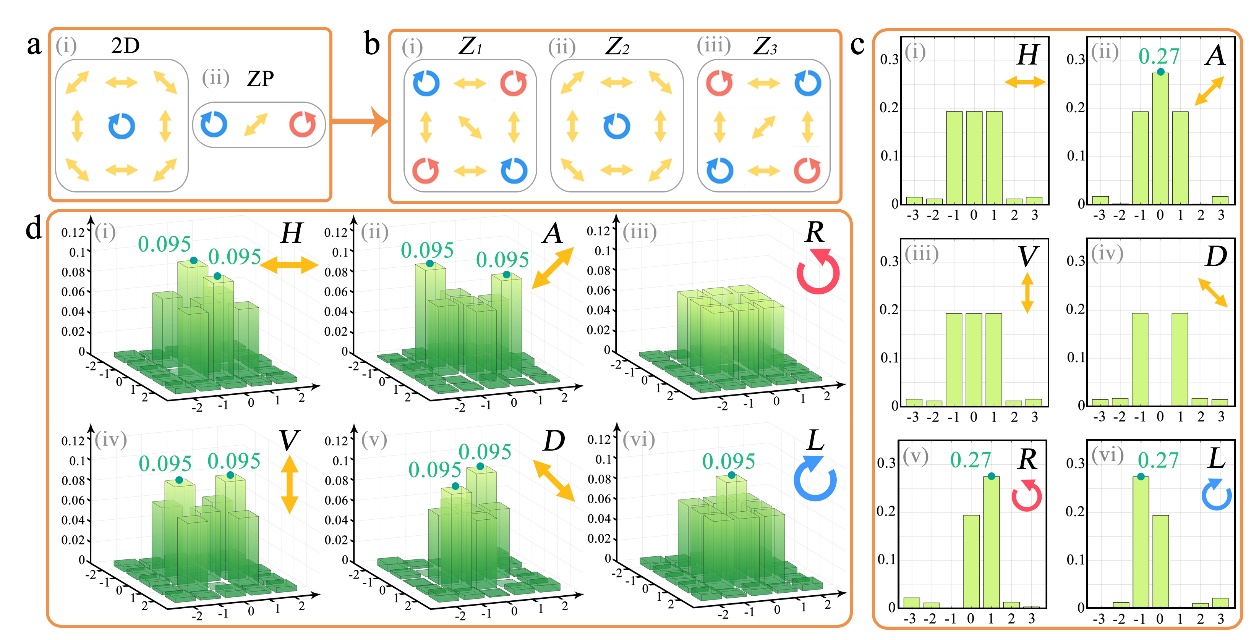


**Fig. S7. Energy distribution results of vectorial Dammann optimization for Sample #4.** a. Target polarization of vectorial 2D Dammann optimization and vectorial Dammann zone plate respectively. b. Target polarization of special 3×3×3 diffraction orders. c. Vectorial optimization results for the 3×3 diffraction orders in the plane. d. Vectorial optimization results for the vector Dammann zone plate.


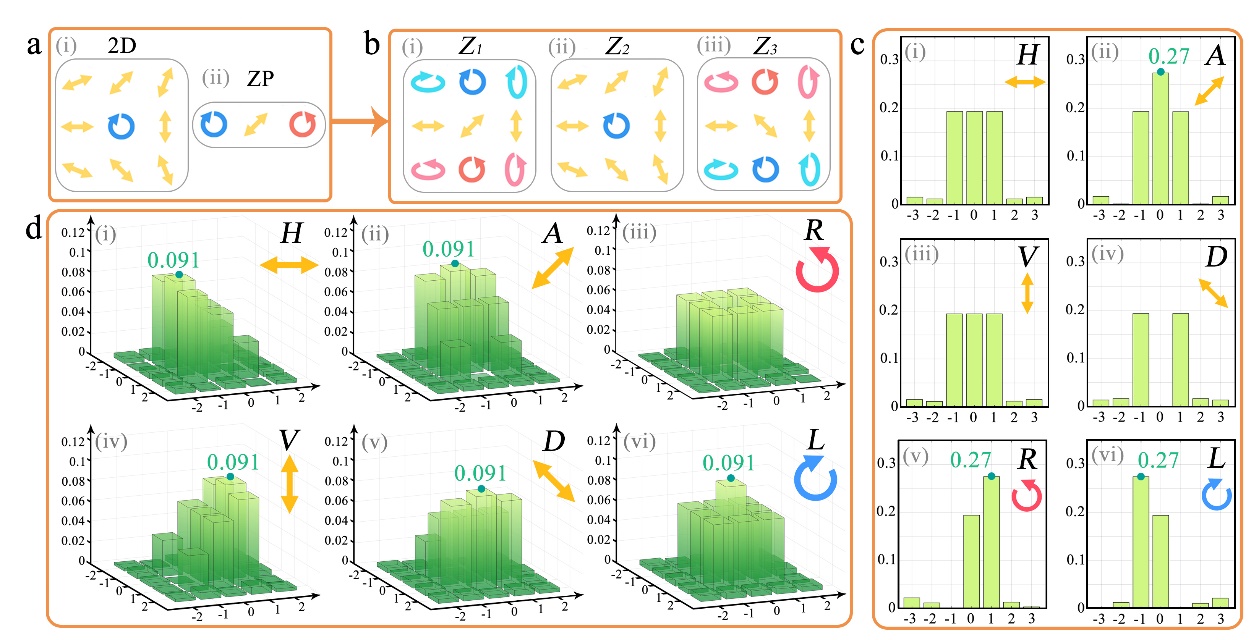


**Fig. S8. Energy distribution results of vectorial Dammann optimization for Sample #5.** a. Target polarization of vectorial 2D Dammann optimization and vectorial Dammann zone plate respectively. b. Target polarization of special 3×3×3diffraction orders. c. Vectorial optimization results for the 3×3 diffraction orders in the plane. d. Vectorial optimization results for the vector Dammann zone plate.


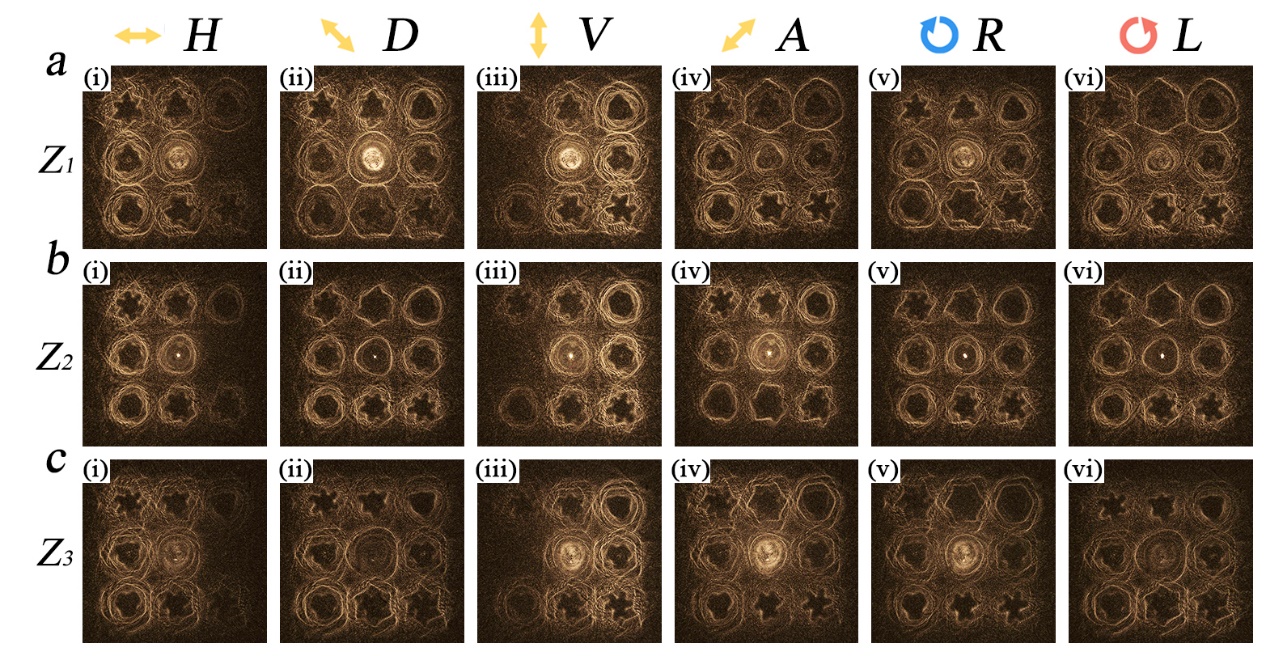


**Fig. S9. Experimental result of vectorial stereoscopic GVB arrays for Sample #5.** The GVB arrays show the desired intensity profiles. Under six typical polarization states, the patterns corresponding to different polarization states appear and hide alternately based on Malus’ principle.


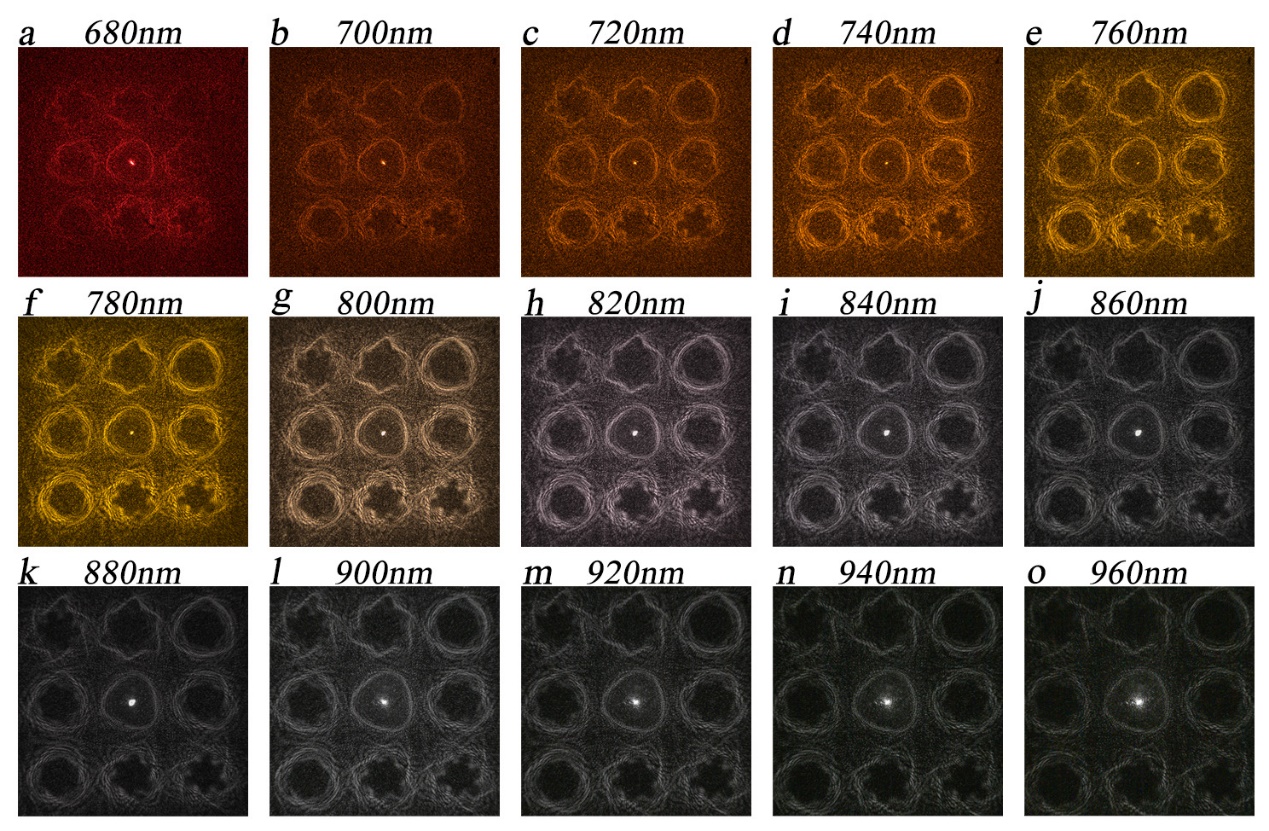


**Fig. S10. Experimental results for broadband verification of vectorial GVB array at different wavelengths ranging from 680 nm to 900 nm for Sample #5.**


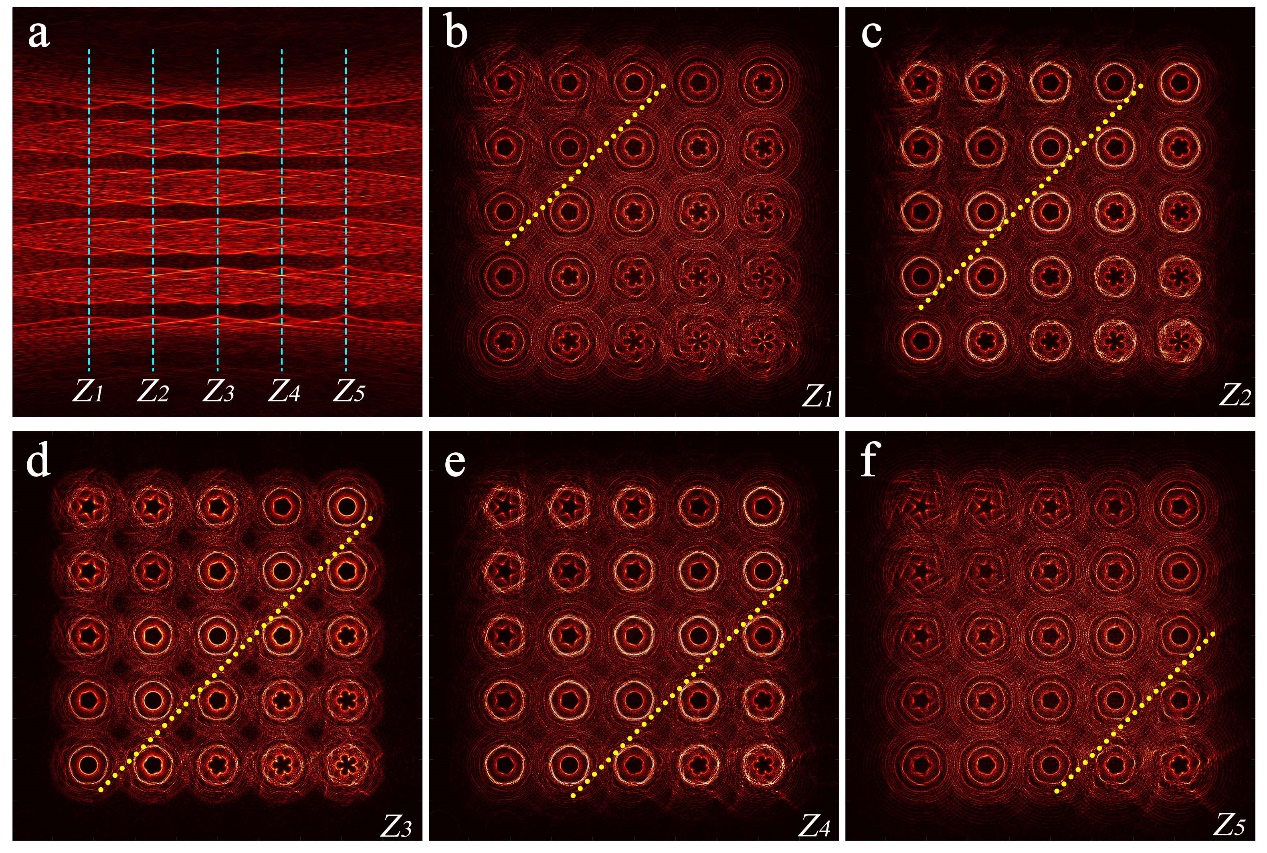


**Fig. S11. Simulation result of scalar 5×5×5 GVB arrays. a.** Simulation results of transmit light propagation in the xz plane at y=0. **b-f.** Simulation results of 2D GVB in each transverse plane, corresponding to Z_1_, Z_2_, Z_3_, Z_4_, and Z_5_ plane, respectively.


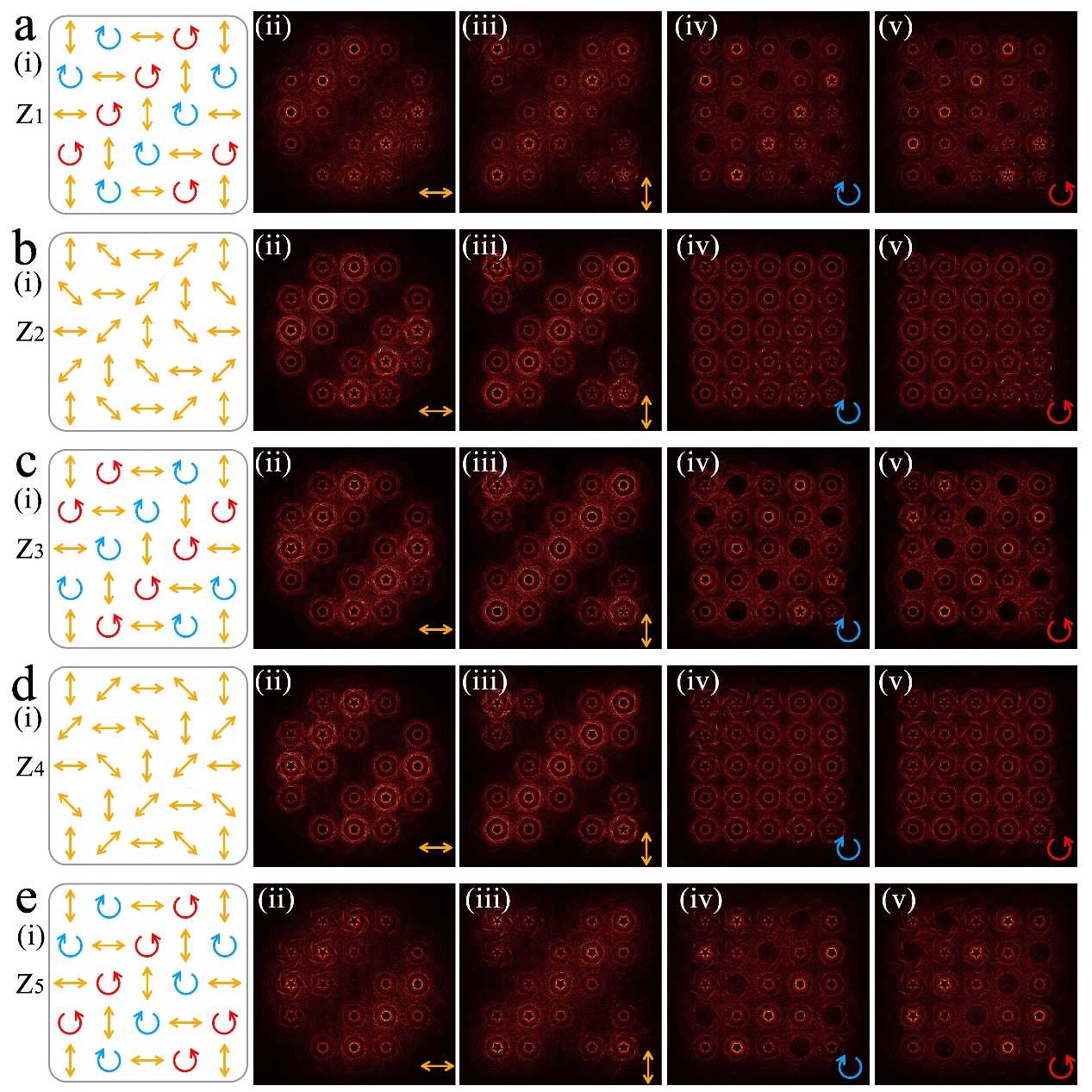


**Fig. S12. Simulation result of vectorial 5×5×5 GVB arrays propagation.** (a-e) The vectorial features, intensity profiles under different polarization analysis are provided in Z_1_ to Z_5_ plane, respectively.

**\**

1. Email: chengwei.qiu@nus.edu.sg [↑](#footnote-ref-1)
2. Email: shuzhang@hku.hk [↑](#footnote-ref-2)
3. Email: huanglingling@bit.edu.cn [↑](#footnote-ref-3)
